# Supplementary material for: Integrin-Linked Kinase (ILK) Promotes Mitochondrial Dysfunction by Decreasing CPT1A Expression in a Folic Acid-Based Model of Kidney Disease
Source: Int J Mol Sci. 2025 Feb 21;26(5):1861. doi: 10.3390/ijms26051861 (PMC11899702; doi:10.3390/ijms26051861)
Supplement: Supplementary file 1 [file ijms-26-01861-s001.zip › ijms-3456324-supplementary.pdf]

Integrin-linked kinase (ILK) promotes mitochondrial dysfunction by decreasing CPT1A expression in a folic acid-based model of kidney disease

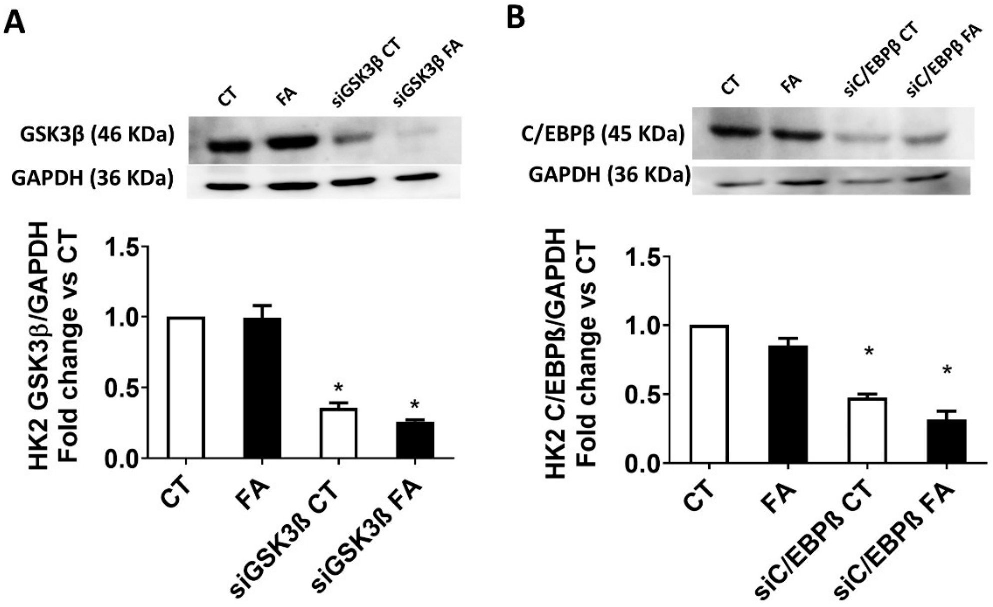

Figure S1: silencing GSK3β and C/EBPβ on HK2 cells. HK2 cells were transfected for 24h with a specific siRNA against ILK GSK3β (siGSK3β), C/EBPβ (siC/EBPβ) or scramble siRNA as transfection control and treated afterwards with 10 mM FA or vehicle (CT) for another 24h. A) Total GSK3β protein content from siILK- siGSK3β-transfected cells from 3 independent experiments, and B) total C/EBPβ protein content from siC/EBPβ-transfected cells, were all determined by western blot. Protein densitometries were normalized to GAPDH, from 5 independent experiments. Relative fold changes vs CT are represented. The results are expressed as fold change of CT and are the mean ± SEM. *$p < 0.05$ vs CT.
